# Toward a Unified Understanding: A Literature Review of Self-Care Conceptualization for the General Population

**DOI:** 10.34172/hpp.45126

**Published:** 2026-06-06

**Authors:** Alexandra Marques-Pinto, Sofia Oliveira, Karina Moutinho, Mirela Ricarte, Carla Zambaldi, Sirley Almeida, Pompéia Villachan-Lyra, Ana Maria Fonte Alves, Taciana Breckenfeld, Maria Carolina Marques dos Santos, Joana Sampaio de Carvalho, Ari Gómez-Borges

**Affiliations:** ^1^CICPSI, Faculdade de Psicologia, Universidade de Lisboa, Lisbon, Portugal; ^2^Business Research Unit (BRU), ISCTE - Instituto Universitário de Lisboa, Lisbon, Portugal; ^3^Universidade Federal de Pernambuco, Recife, PE, Brazil; ^4^Universidade Federal Rural de Pernambuco, Recife, PE, Brazil; ^5^WANT Research Team, Universitat Jaume I, Castelló de la Plana, Spain

**Keywords:** Concept analysis, General population, Literature review, Self-care, Self-care theory

## Abstract

**Introduction:**

The study and conceptualization of self-care have a longstanding history. However, a clear conceptualization of self-care remains contested due to the plurality of perspectives and diversity of definitions, particularly when considering the general population regardless of pathology. Our literature review aimed to better understand how to conceptualize self-care for the general population, drawing on existing scientific literature on self-care theory and conceptualization.

**Methods:**

We followed The Cochrane Collaboration’s and PRISMA-P Group’s methodological guidelines for the conceptualization, analysis, and reporting of literature reviews.

**Results:**

Our results reaffirmed the varied and evolving interpretations of self-care and provided insights into conceptualizing and promoting self-care among the general population. The analysis, based on three main criteria (definitions, antecedents, and consequences), highlighted a continuous renewal of the self-care concept across various disciplines and demographic groups. For the general population, self-care is fundamentally intentional and purpose-driven, encompassing a wide spectrum of purposes from health management to enhancing overall well-being. This multifaceted concept integrates actions, abilities, and socio-cognitive processes and emphasizes the importance of understanding the multiple antecedents of self-care to develop effective policies and interventions that enhance well-being and quality of life across diverse groups.

**Conclusion:**

From our perspective, the conceptualization of self-care for the general population benefits from a systemic, ecological view. Moreover, promoting self-care outcomes, such as autonomy, independence, and self-efficacy, can yield benefits, including improved health and well-being for the general population. We advocate for a multi-tiered approach to self-care promotion, implemented through schools, workplaces, and broader community contexts, to maximize benefits across society.

## Introduction

 This study aimed to conduct a literature review (also known as a narrative review^1^) of self-care theory and conceptualization, considering its historical origins and evolution. Our goal was to better understand how self-care can be conceptualized for the general population. The term ‘general population’ is used herein to describe individuals across a spectrum of physical, mental, and social health statuses, spanning from healthy individuals or without diagnosed conditions to those experiencing subclinical conditions, health-related risks, or challenges related to their well-being. Given our aim of tracing the development of self-care theory across various periods and intellectual contexts, a literature review was considered the most appropriate methodological approach,^2^ enabling a contextualized synthesis of diverse sources and a comprehensive understanding of the concept’s development.^1,2^

 Self-care is a difficult concept to frame outside of the healthcare domain, as its conceptualization and study have been closely associated with research and intervention in the field of health/disease. Historically, self-care emerged as a set of practices and behaviors that allowed people to maintain their health and deal with situations of illness.^3^ With the development of modern medicine, particularly in the 19th century, (health)care became increasingly professionalized and focused on acute treatment. However, in the 2^nd^ half of the 20^th^ century, the prevalence of chronic diseases (e.g., diabetes, chronic pain)^4^ renewed the relevance of self-care, emphasizing individual autonomy in these conditions’ prevention and management.^3^ Foundational contributions include Orem et al,^5^ who defined self-care as the practice of activities that individuals perform for their benefit, to maintain life, health, and well-being. Similarly, Levin, who is considered the “father” of self-care, defined self-care as a process through which lay people take ownership of the promotion of their health as well as the prevention, detection, and treatment of their illnesses.^6^

 Over time, attention expanded to self-care among healthcare service professionals^7^ (e.g., nurses,^8^ physicians,^9^ psychotherapists^10^), and more broadly to human services professionals (e.g., social workers^11^ and teachers^12^). For these professionals, self-care is often regarded as both an ethical responsibility^13^ and a means of promoting occupational health and well-being.^8,11^ Thus, in these fields, self-care has been incorporated into initial professional training,^14^ and under the influence of Positive Psychology, increasingly framed as a means of promoting workers’ positive well-being as an end.^15^ Nonetheless, gaps remain regarding the self-care of professionals outside of human service fields.^15^

 A second challenge in conceptualizing self-care for the general population pertains to the plurality of the self-care concept. The term has been associated or related to a variety of overlapping constructs, such as “caring of oneself”, “self-help”, “self-agency”, “self-efficacy”, “self-management”, “self-monitoring”, “self-treatment”, “activities of daily living”,^3,16^ and is characterized by substantial definitional and theoretical diversity. For instance, Godfrey et al^3^ identified over a hundred different definitions of self-care, varying across academic fields. While a common definition may be possible, according to Levin et al, ^6^ it would also come at the cost “of losing the precision required for significant analytic research” (p. 10). Nevertheless, this heterogeneity hinders self-care operationalization and research.^3^

 Diversity is also reflected in the approaches adopted to characterize and explain self-care. Some studies adopt predefined theoretical perspectives as an *a priori* frame of reference (e.g., Cognitive Behavior Model,^17^ Emotional Intelligence Theory ^18^), whereas others follow more inductive approaches. Recent work have conceptualized self-care through three key descriptors (i.e., antecedents, attributes, and consequences).^19^ Building on this perspective, El-Osta et al^20^ have identified multiple theoretical models and organized them into an integrative systemic model – the *Matrix of Self-Care* –, which structures self-care across four dimensions: activities, behaviors, context, and environment.

 Additionally, self-care is often conceptualized as multidimensional, encompassing various dimensions, such as physical, psychological, social, and spiritual. While this broad spectrum may be relevant across diverse populations,^21^ research has predominantly focused on clinical groups (e.g., diabetes) or high-risk populations (e.g., healthcare professionals). More recently, some studies have begun to address self-care in underexplored groups, including children ^22^ and healthy individuals,^21^ highlighting the broader applicability of self-care.

 Although significant research has broadened our understanding of self-care across varied groups, there remains a lack of a clear conceptualization of self-care for the general population. This limitation constrains the development of robust assessment tools, contributes to the scarcity of large-scale studies, and hinders the design of effective public policies and interventions.^23^ Without such tools and solid empirical evidence on which dimensions should be targeted for change and how they relate to health and well-being outcomes, efforts to promote self-care in the general population remain compromised.^21^

 To address this gap, this literature review synthesizes the conceptualization and theories of self-care as they apply to the general population, considering their historical evolution. By doing so, it aims to support the organization of key self-care markers applicable to the general population; foster a shared interdisciplinary language for researchers and practitioners; and contribute to the development of a coherent conceptual framework to guide the assessment and design of policies and targeted interventions that leverage self-care to improve health and well-being in the general population.

## Methods

 We followed The Cochrane Collaboration’s^24^ and PRISMA-P Group et al.’s ^25^ guidelines for literature reviews in the conceptualization of the study, analysis of data, and reporting of results.

###  Search Strategy and Outcomes

**Figure 1:**
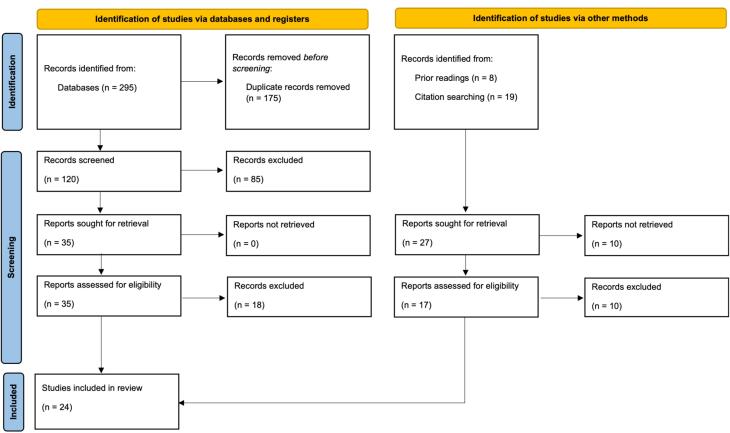
PRISMA Flow Diagram Displaying the Data Collection Process

 Records were primarily searched using advanced and Boolean search options on the *EBSCOhost web*, *SCOPUS*, *SciELO*, and *PubMed* databases on June 28, 2022. The following search terms were cross-searched within the title and/or abstract of the publications: (self care OR self-care OR selfcare) AND (history OR historical OR background OR evolution OR progression OR conceptualiz * OR concept analysis OR concept mapping OR concept OR meaning OR framework OR theory OR model OR review). Studies identified in prior readings from the initial stages of the literature review that complied with the eligibility criteria (see Table 1) were also included in the final pool of records. Additionally, after the database search, reference lists of the identified records were hand searched. Both these complementary strategies allowed for minimizing bias and maximizing the identification of relevant studies.^26^ Figure 1 depicts the PRISMA flow diagram of search results.

**Table 1:** Inclusion and Exclusion Criteria

**Inclusion criteria**	**Exclusion criteria**
Contained key search words	Did not contain key search words
Concerns an historic / conceptual analysis of the self-care concept towards a general population	Papers in which the sample was defined exclusively by having a specific ill-health / pathology (e.g., diabetes).
Full-text version available	Full-text version unavailable
Peer reviewed research with qualitative or mixed-methods design, theoretical, conceptual or review studies	Papers related to the development and/or validation of instruments to assess self-care; Empirical research on the efficacy of self-care interventions; Empirical research measuring self-care (e.g., practices, use, impacts) that did not intend to discuss self-care conceptualization, theorization or definition
	Grey literature, thesis, books, testimonies / comments, or non-peer reviewed papers

 Initial database search yielded 120 non-duplicated records, which were screened against the eligibility criteria, along with the 8 prior studies and 19 hand-searched hits. No language, field of research, or date of publication constraints were applied. As depicted in Figure 1, the initial title/abstract screening led to the exclusion of 85 records that did not meet the eligibility criteria. Then, the full-text screening of the remaining 35 database-identified records and the 27 manually added records led to a total of 24 final records (17 from database search, and 7 hits added via other methods). These 24 eligible records, which met all the selection criteria, were included in the study (see Table S1 in supplementary material).

###  Data Synthesis

 The 24 included records were read in full and synthesized based on the target population for self-care and on three general criteria commonly used in previous literature reviews on self-care^19^: 1) Definition/attributes (i.e., core concepts and characteristics that define self-care); 2) Antecedents(i.e., pre-existing factors or conditions that facilitate or hinder the practice of self-care); and 3) Consequences/results (i.e., outcomes or effects associated with the practice of self-care) (see Table 2). Data were primarily synthesized by five coders. Then, to ensure the quality of the coding process, interrater agreement was computed. This was achieved using a fully crossed design involving an independent coder for 20% of the eligible records. Coding differences were discussed, and studies were re-coded as necessary until full agreement was reached. Cohen’s kappa variant for multiple coders was computed to evaluate the reliability regarding the criterion variables.^27^ A mean kappa value of 0.76 was obtained among the coders, indicating substantial agreement.^27^ Kappa values between each pair of coders ranged from 0.54 to 1.00, indicating moderate to perfect agreement.

**Table 2:** Self-care Target Population, Definitions/Attributes, Antecedents and Consequences/Results

**Code**	**Authors**	**Target Population**	**Definitions**	**Antecedents**	**Consequences**
1	Bressi & Vaden ^28^	General population (Professionals)	x		x
2	Chipu & Downing ^29^	General population (Professionals)	x	x	x
3	El-Osta et al. ^20^	General population			x
4	Gantz ^30^	General population and Patients	x	x	x
5	Gast et al. ^31^	General population (Adolescents)	x	x	
6	Godfrey et al. ^32^	General population	x	x	x
7	Godfrey et al. ^3^	General population (Professionals)	x	x	x
8	Holguín-Lezcano ^10^	General population (Professionals)	x	x	x
9	Horowitz ^33^	General population	x	x	x
10	Høy et al. ^34^	General population (Elders)	x	x	x
11	Jones et al. ^35^	General population and Patients	x	x	x
12	Lee & Miller ^36^	General population (Professionals)	x	x	x
13	Levin & Idler ^37^	General population (Professionals)	x		x
14	Lommi et al. ^38^	General population (Professionals)		x	x
15	Mailhot et al. ^39^	General population and Patients	x	x	x
16	Martínez et al. ^19^	General population (Professionals) and Patients	x	x	x
17	Marzband & Zakavi ^40^	General population	x	x	x
18	Matarese et al. ^41^	General population	x	x	x
19	McCormack ^42^	General population (Professionals)	x	x	x
20	Miller et al. ^43^	General population (Parents)	x	x	x
21	Richard & Shea ^44^	General population	x	x	x
22	Tulu et al. ^45^	General population (Professionals)	x	x	x
23	Wilkinson & Whitehead ^46^	General population and Patients	x	x	
24	Woods ^47^	General population	x		x

 After extracting the qualitative data synthesized within the three general criteria for conceptualizing self-care, an inductive thematic content analysis was conducted, and a category system was developed for each criterion. Given their cyclical and dynamic nature, the general stages of this technique were followed.^48,49^ Once the corpus of analysis had been constituted, we performed an active and progressively in-depth reading and re-reading process to enhance our comprehension of the data and develop familiarity with the content. We followed an open coding procedure. Categories were primarily created by systematically aggregating recording units with a common meaning. These categories were then revised and modified (agglomerated or divided into sub-categories) until the consolidated category system was reached. For example, within the theme *Personal/internal factors* (self-care antecedents), the subcategory *awareness of imbalance* (*n* = 10) was generated inductively by grouping codes such as ‘judgment of imbalance’, ‘imbalance perception’, and ‘difficulty in setting boundaries’. This subcategory was then merged with other conceptually related subcategories, such as ‘self-efficacy’, ‘self-esteem’, ‘confidence’, ‘acceptance of responsibility’, and ‘skills/capacities’, to form a broader category labelled *Specific *(personal/internal factors). This was distinguished from a separate category labelled *General *(personal/internal factors), which aggregated more global psychological and emotional, cognitive, and demographic characteristics. To ensure the quality of the categorization, we considered the following criteria: mutual exclusion, exhaustivity, homogeneity, relevance, objectivity, and productivity.^48^ This process was primarily conducted by one researcher, with the support of four other researchers. Subsequently, 25% of the corpus was independently rated by two judges to ensure the reliability of the category system. Discrepancies were analyzed and resolved by reviewing and readjusting the system until 100% agreement was reached.

 Regarding the quality assessment of the eligible studies, our record pool exclusively includes conceptual and theoretical studies. Thus, formal empirical quality appraisal frameworks (e.g., CASP, SPIDER) were not applicable. Instead, although formalized quality appraisal procedures are not mandatory in literature reviews,^50^ we evaluated the conceptual rigor of the included papers, ensuring they depicted their aim/purpose, were transparent and coherent, and had an overall theoretical contribution. For studies that developed concepts through structured literature analyses, we applied an adapted CAMELOT-informed assessment.^51^ We analyzed meta-domains (i.e., disclosure of research aims, funding and/or conflicts of interest, and author involvement), research design domains (i.e., research strategy, inclusion criteria, and theoretical grounding), and research conduct domains (i.e., procedures for analyzing and interpreting data, and transparency in presenting findings). Studies meeting these criteria were mostly considered to present minimal or minor concerns (see Table S2 in supplementary material), thereby ensuring transparency and minimum standards of rigor. Three studies were evaluated as presenting moderate or unclear concerns due to the omission of information. However, since our study aimed to map the conceptualization of self-care for the general population, we prioritized providing a thorough range of definitions over excluding studies based on quality. As this research did not involve human subjects, it was exempt from Ethical and Deontological Board approval.

## Results

Table 2 depicts, for the 24 articles analyzed, their target population (e.g., the general population, specific subgroups within it) and whether they presented information on the three general criteria considered for the analysis. All 24 papers were included in the analysis of the self-care concept.

###  Self-Care Definitions/Attributes

 Of the 24 articles analyzed, 22 defined self-care (Table 2), revealing four main themes: (1) Purposes of self-care, such as the promotion, maintenance, and management of health and well-being (*N* = 32, e.g., “…to promote the physical, mental, emotional and spiritual being…” - ^29^ pp. 451), as well as the management, prevention, and recovery from illnesses (*N* = 19, e.g., “…managing acute and chronic conditions…” - ^41^ pp. 302); (2) Dimensions, including actions (*N* = 13, e.g. “… sleep hygiene, social support, …” - ^44^ pp. 107), skills, processes and endeavors; (3) Agents and Recipients, where self-care can be performed by individuals (*N* = 16) for themselves (e.g., “… to care for oneself to preserve one’s own health…” - ^29^ pp. 451), for others, or for the environment, with different levels of autonomy (e.g., “... individuals are informed by technical knowledge and skills derived from the pool of both professional and lay experience…” - ^37^ pp. 181), within a given social and cultural context; and (4) Influences (*N* = 3), encompassing situational and cultural factors, knowledge, skills, values, and motivation (see Table 3).

**Table 3:** Definitions/Attributes of Self-care: Themes, Categories, and Subcategories of Core Concepts and Characteristics that Define Self-care

**Theme**	**Categories subcategories**	**Frequency**	**Examples (references)**
Purpose: With what purpose is self-care carried out?	*Health and well-being*	*N* = 32	
	promote/achieve	*n* = 16	“…to promote the physical, mental, emotional and spiritual being…” (Chipu & Downing^29^, pp. 451) “… in order to achieve… optimal health and well-being…" (Martínez et al. ^41^, pp. 422)
	sustain (maintain)	*n* = 10	“…sustaining holistic health and well-being (Lee & Miller^36^, pp. 98) “… directed toward maintaining health…” (Matarese et al. ^41^, pp. 298)
	manage (regulate, control)	*n* = 6	“… actions of the person to manage their health …” (Jones et al. ^35^, pp. 181) “… in order to regulate one’s functioning in the interest of one's life, integrated functioning and well-being…” (Gast et al.^31^, pp. 27);
	*Illness or disability*	*N* = 19	
	manage	*n* = 8	“…managing acute and chronic conditions…” (Matarese et al. ^41^, pp. 298)
	prevent	*n* = 6	“…this includes taking action to prevent illness and accidents…” (Wilkinson & Whitehead^47^, pp. 1145)
	recover (remedy, treat)	*n* = 5	“…recovery… from experiences of illness and injury…” (McCormack^42^, pp. 51)
Dimensions: How is self-care carried out?	*Action/Activity/behavior*	*N* = 13	“… sleep hygiene, social support, …” (Miller et al. ^43^, pp. 107)
	*Ability, capacity or skill*	*N* = 8	“… awareness, self-control, and self-reliance…” (Martínez et al. ^41^, pp. 422)
	*Process*	*N* = 5	“… a process of purposeful engagement…” (Lee & Miller^36^, pp. 98)
	*Endeavor/attempt/effort*	*N* = 3	“…dynamic, purposeful, and continuous endeavor essential for all human beings…” (Tulu et al.^45^, pp. 5)
Agent and Recipient: By / to whom is self-care carried out?	*By an individual*	*N* = 16	
	for his/her own health	*n* = 13	“… to care for oneself to preserve one’s own health…” (Chipu & Downing^29^, pp. 451)
	for other people health and environment	*n* = 3	“... is performed for … other…” (Tulu et al. ^45^, pp. 5) “…actions directed toward… the environment… (Gast et al. ^31^, pp. 27)
	*Autonomy gradient*	*N* = 6	
	with advice, support or informed by others	*n* = 4	“... individuals are informed by technical knowledge and skills derived from the pool of both professional and lay experience…” (Levin & Idler^4^, pp. 181)
	an autonomous practice	*n* = 2	“… actions decided autonomously…” (Matarese et al. ^41^, pp. 299)
	*The social and cultural context*	*N* = 3	“…a social and cultural context is required…” (McCormack^42^, pp. 49)
Influences: Under what influence is self-care carried out?	*Different variables and processes*	*N* = 3	“… is influenced by knowledge, skills, values, motivation, locus of control, and efficacy…” (Gantz^30^, pp. 2)

*Note*. Frequency - count of occurrences of each category or subcategory in the 24 articles considered.

###  Self-Care Antecedents 

 The authors of 20 articles identified two main themes of antecedent factors (Table 2). The first theme refers to Personal/Internal factors and includes specific elements (*N* = 72) such as skills (e.g., “… Self-care is influenced by internal factors as… commitment…” - ^41^ pp. 298) or self-regulation. It also includes general factors (*N* = 6) such as psychological factors (e.g., “…structures of support for personal care… b) psychological and emotional…” - ^36^ pp. 100) and demographic aspects. The second main theme relates to Interpersonal/External specific factors (*N* = 15) and covers different types of support (e.g., “…availability of social support” - ^41^ pp. 298; “…professional care: … d) professional social support - ^36^ pp. 100) as well as resources. Within this theme, general factors (*N* = 13) refer to social and cultural contexts (e.g., “… social and cultural contexts…” - ^34^ pp. 461) (see Table 4).

**Table 4:** Antecedents of Self-care: Themes, Categories, and Subcategories of Pre-existing Factors or Conditions that Facilitate or Hinder the Practice of Self-care

**Theme**	**Categories subcategories **	**Frequency**	**Examples (references)**
Personal / internal factors.	*Specific*	*N* = 72	
	skills or capacities	*n* = 20	“… an individual’s ability to carry out self-care is directly related to his or her … skill…”(Martínez et al.^41^, pp. 421) “… self-care is influenced by internal factors as… commitment…” (Matarese et al. ^41^, pp. 298)
	acceptance of responsibility	*n* = 11	“…acknowledging responsibility for one’s own life and the decisions one makes…” (Godfrey et al. ^3^, pp. 8)
	awareness of imbalance	*n* = 10	“… barriers… difficulties in setting boundaries…” (Miller et al. ^41^, pp. 109) “… la présence d’un déséquilibre…” (Mailhot et al. ^39^, pp. 99)
	self-efficacy/ esteem/confidence	*n* = 8	“…the sense of self-efficacy, self-esteem… in the process that leads to decide which care activities to undertake for himself…” (Lommi et al.^38^, pp. 159)
	values and beliefs	*n* = 8	“… religious and cultural beliefs…” (Chipu & Downing^29^, pp. 450)
	motivation	*n* = 6	“…individual will and motivation…” (Godfrey et al. ^3^, pp. 8) “… an individual’s ability to carry out self-care is directly related to his or her... motivation …”(Martínez et al. ^41^, pp 421)
	knowledge and learning	*n* = 5	“… they must also have a found of knowledge about self-care…” (Gast et al. ^31^, pp. 37) “... en effet, dix antécédents sont bien distingués par les auteurs en sciences infirmières, soit l’apprentissage…” (Mailhot et al. ^39^, pp. 99)
	self-regulation/control/agency	*n* = 4	“… was influenced by … self-control…” (Martínez et al.^41^, pp. 420)
	*General *	*N* = 6	
	psychological or emotional	*n* = 3	“…structures of support for personal care… b) psychological and emotional…” (Lee & Miller^36^, pp. 100)
	cognitive	*n* = 2	“… patient-related antecedents include … cognition …” (Martínez et al.^41^, pp. 421)
	demographic	*n* = 1	“… antecedents of self-care include … demographic… factors (Richard & Shea ^44^, pp.256) “… It is also influenced by age…” (Lommi et al. ^38^, pp. 159)
Interpersonal and external factors	*Specific *	*N* = 15	
	Support:	*N* = 10	
	social	*n* = 5	“…availability of social support” (Matarese et al.^41^, pp. 298)
	religious or spiritual	*n* = 2	“…spiritual … support…” (Chipu & Downing^29^, pp. 450)
	professional	*n* = 2	“…professional care: … professional social support (Lee & Miller^36^, pp. 100)
	mutual aid	*n* = 1	“… self-care was associated with two other mechanisms: mutual aid...” (McComarck^42^, pp. 53)
	Resources:	*N* = 5	
	of the healthcare system	*n* = 2	“… healthcare approaches…” (Høy et al. ^34^, pp. 461)
	other	*n* = 3	“…time, limited money…” (Miller et al. ^43^, pp. 109)
	*General*	*N* = 13	
	social and cultural	*n* = 4	“… social and cultural contexts…” (Høy et al. ^34^, pp. 461)
	physical environment	*n* = 3	“… self-care is … environmental…” (Godfrey et al. ^32^, pp. 9)
	political and economic	*n* = 2	“… impacted by … economic and political factors…” (Wilkinson & Whitehead^46^, pp. 1146)
	resources	*n* = 2	“… access to adequate resources” (Martínez et al.^41^, pp 421)
	other	*n* = 2	“…structures of support for personal care… leisure…” (Lee & Miller^36^, pp. 99)

*Note*. Frequency - count of occurrences of each category or subcategory in the 24 articles considered.

###  Self-Care Consequences/Results

 Out of the 24 articles analyzed, 22 discussed the consequences of self-care (Table 2). All of those studies, except for one,^34^ highlighted beneficial outcomes of self-care within four main themes. Self-care was described as contributing to: (1) Learning and Skills Development for both patients (*N* = 23, e.g., “… to reach autonomy…” -^41^ pp. 298) and professionals (*N* = 6, e.g., “…thrive in the context of work environments that involve continued and prolonged exposure to oppression and human suffering…” - ^28^ pp. 37); and improved (2) Health and Well-being, specifically by increasing, maintaining and promoting health and well-being (*N* = 14, e.g., “…physical, mental and social well-being…” - ^34^ pp. 462) along with more general positive outcomes such as acquiring social support. Additionally, self-care was associated with beneficial consequences regarding (3) Disease, such as prevention, detection, and treatment (*N* = 8, e.g., “… prevention of diseases and accidents…” - ^35^ pp. 178); and reduction of (4) Occupational Health problems (*N* = 5), namely stress (e.g., “…reduced occupational stress…” - ^16^ pp. 420), burnout, and turnover. Notably, Høy et al.^34^ acknowledged both positive and negative outcomes, for example, “…helplessness, apathy, dependency and even abandoned self-care…” (pp. 462), which could lead to responsibility overload or professional care neglect (see Table 5).

**Table 5:** Consequences/Results of Self-care: Themes, Categories, and Subcategories of Outcomes or Effects Associated with the Practice of Self-care

**Theme**	**Categories subcategories **	**Frequency**	**Examples (references)**
Beneficial consequences regarding learning and skills development	*For healthcare system users*	*N* = 23	
	autonomy and independence	*n* = 8	“… to reach autonomy…” (Matarese et al. ^41^, pp. 298)
	experience empowerment	*n* = 3	“…empowerment…” (Chipu & Downing^29^, pp. 450)
	self-esteem and self-confidence	*n* = 3	“… positive self-esteem…” (Høy et al. ^34^, pp. 459)
	preserve and transcend the self	*n* = 3	“…transcendence of self …” (Høy et al. ^34^, pp. 459)
	acquire knowledge and skills	*n* = 2	“…acquisition of new knowledge and skills…” (McCormack^42^, pp. 56)
	other	*n* = 4	“…dominar sus propios temores …” (Holguín-Lezcano et al. ^10^, pp. 161)
	*For health professionals*	*N* = 6	
	personalize the intervention / management of patients' symptoms / illnesses and disabilities	*n* = 4	“… specific interventions to individualize care toward achieving the most relevant goals…” (Richard & Shea^44^, pp. 255)
	other	*n* = 2	“…thrive in the context of work environments that involve continued and prolonged exposure to oppression and human suffering…” (Bressi & Vaden^28^, pp. 5)
Beneficial consequences regarding health and well-being	*Specific *	*N* = 14	
	Increasing, maintaining, promoting:	*N* = 14	
	one's own health /mental health	*n* = 9	“…for their own healthy development…” (Horowitz^33^, pp. 60)
	one's own well-being	*n* = 3	“…physical, mental and social well-being…” (Høy et al.^34^, pp. 462)
	the health of patients	*n* = 2	“…better patient health outcomes…” (Tulu et al. ^45^, pp. 7)
	*General*	*N* = 8	
	acquiring social support	*n* = 3	“…self-care also involves numerous positive consequences and effects, such as…a sense of social support…” (Marzband & Zakavi^40^, pp. 4)
	making lifestyle changes	*n* = 2	“…changes in lifestyle…” (Levin & Idler^4^, pp. 194)
	other	*n* = 3	“…a pure life…” (Marzband & Zakavi^40^, pp. 4)
Beneficial consequences regarding disease	*Prevention, detection, and treatment of diseases / accidents *	*N* = 8	“… prevention of diseases and accidents…” (Jones et al. ^35^, pp. 178)
	*Coping with illnesses *	*N* = 5	“…enhanced coping…” (Martínez et al. ^41^, pp. 421)
	*Decrease in morbidity / elimination of symptoms*	*N* = 2	“…la suppression des symptômes…” (Mailhot et al. ^39^, pp. 101)
	*Recovery / restoration of health after illness*	*N* = 2	“…recovery from minor ailments…” (Jones et al. ^35^, pp. 182)
	*Reduction of mortality *	*N* = 2	“…lower mortality…” (Tulu et al.^45^, pp. 6)
	*Other*	*N* = 2	“…increased stability of illness…” (Tulu et al.^45^, pp. 7)
Beneficial consequences regarding occupational health	*Reduction of occupational stress symptoms*	*N* = 2	“…reduced occupational stress…” (Martínez et al.^41^, pp. 420)
	*Alleviation of burnout*	*N* = 2	“…burnout prevention…” (Miller et al. ^43^, pp. 110)
	*Decrease in turnover*	*N* = 1	“…turnover…” (Lee & Miller^36^, pp. 101)
Negative consequences	*Adverse effects*	*N* = 1	“… absence of social support had negative consequences for… well-being…” (Høy et al.^34^, pp. 462)

*Note*. Frequency - count of occurrences of each category or subcategory in the 24 articles considered.

## Discussion

 This study aimed to conduct a literature review on self-care theory, tracing its historical roots and evolution to understand its conceptualization for the general population, here defined as the population at large, assumed to be healthy within a broad spectrum of physical, mental, and social health statuses, or at least without a known condition of illness. Although using this definition of the general population to establish the eligibility criteria for studies included in the literature review, results show that some articles discuss self-care in both the general population and among individuals with chronic or acute diseases. Additionally, among the specific subgroups within the general population, professionals (vs. non-professionals) outnumbered other subgroups, such as those defined by age (comparing children, adolescents, adults, and/or elderly) (see Table 2). The prevalence of studies focused on professional groups, compared with other groups of the general population, suggests that self-care remains primarily framed as a response to occupational or health risks, rather than as a universal resource for everyday well-being. These findings may reflect a trend seen in the historical conceptualization and study of self-care, which has been closely linked to knowledge and interventions targeting groups with certain conditions (e.g., diabetes patients) or risk factors (e.g., healthcare professionals).^3,7,11^ However, our results on self-care, examined through the three common criteria used in previous literature reviews (i.e., definition/attributes, antecedents, and consequences), highlighted the complex nature of self-care and provided valuable insights into how to conceptualize and promote self-care within the general population, as described below.

**Figure 2:**
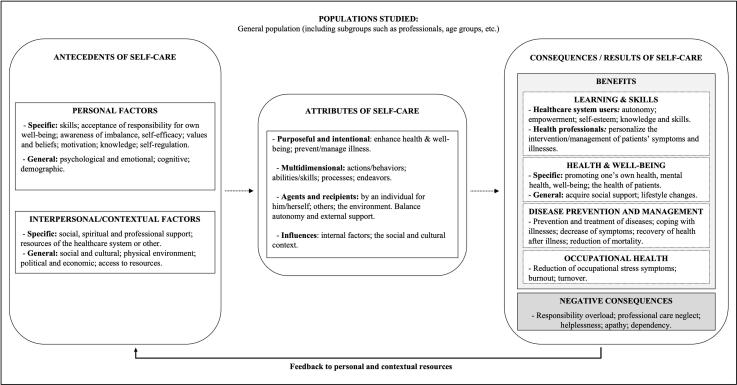
Conceptual Model of Self-care for the General Population, Depicting Self-care Antecedents, Attributes, and Consequences

Figure 2 presents a conceptual model of self-care for the general population derived from our review. On the left, it groups the antecedents of self-care into personal factors and interpersonal or contextual influences. In the center, the figure displays the core attributes of self-care in the general population, emphasizing its purposeful and intentional nature, its multidimensional character, and its orientation toward the self, others, and the environment. On the right, it summarizes the consequences of self-care, including benefits related to learning and skills development, health and well-being promotion, disease prevention and treatment, and occupational health, as well as potential negative consequences (e.g., when responsibility is shifted excessively to individuals without adequate professional support). The arrows indicate the directional links from antecedents to attributes and from attributes to consequences, and a feedback arrow from consequences back to antecedents, highlighting how self-care processes and outcomes can, over time, reshape the personal and contextual conditions that support self-care. These findings are discussed further in the following sections.

###  Self-Care Definitions/Attributes

 The analyzed definitions generally conceptualize self-care as purposeful and “intentional (…), rather than reactive and ad hoc”.^36^ This reveals a spectrum of purposes for self-care (see Table 3), ranging from the prevention and management of illnesses and disabilities to the enhancement of health and well-being. This understanding underscores the importance of the general population taking a proactive approach to their health. Awareness, along with other prerequisites discussed later, emerges as a foundation for effective self-care behaviors. To achieve these objectives through self-care practices, individuals must be aware of their health status, including perceived threats to their health, as well as of effective coping strategies to address these threats.^19^ Similarly, awareness of the benefits and barriers to self-care practices ^52^ can motivate individuals to engage in these practices and develop strategies to overcome potential obstacles. By recognizing the spectrum of purposes of self-care, which extends beyond managing illnesses to include enhancing health and well-being, interventions can be designed to cater for different objectives and stages of health among individuals in the general population. This will boost the effectiveness and sustainability of self-care practices. The diversity of self-care purposes, spanning from health to illness, is reflected in the benefits mentioned in various of the reviewed articles on the consequences of self-care, as will be discussed further ahead.

 Secondly, acknowledging self-care as a multifaceted construct encompassing various dimensions,^35,42,53^ from actions to abilities, processes, and endeavors (see Table 3), allows us to appreciate its complexity among the wider population. Integrating these diverse perspectives enables a comprehensive understanding of self-care. This includes not only the deliberate practices that individuals undertake to sustain their health and well-being (as proposed by Orem et al.’s Self-Care Deficit Theory^5^), but also the underlying variables and processes (e.g., socio-cognitive processes, as described by Bandura^54^) that regulate these actions. Acknowledging the multidimensional nature of self-care and understanding the interplay between actions and related mechanisms allows practitioners to tailor interventions to effectively address the diverse needs and motivations of individuals within the general population.

 Additionally, self-care definitions suggest that it can be carried out by individuals for their health and well-being, although some definitions extend their scope to include others and the environment (see Table 3). Similarly, the findings emphasize the significant influence of social and cultural contexts on the promotion of self-care practices, in line with an ecological perspective.^55^ This highlights the importance of interventions that address the diverse needs of individuals within the general population, while also considering societal factors such as culture, socioeconomic status, social support, and access to healthcare resources, all of which have a significant impact on self-care practices. The interplay between internal factors and external influences on self-care will be discussed in more detail later concerning the findings on the prerequisites for self-care.

 According to our findings, the balance between autonomy and external support may be crucial in promoting effective self-care among the general population. While some definitions portray self-care as an autonomous practice,^35^ others emphasize the importance of external assistance (i.e., from family or professionals) in fostering effective self-care behaviors.^37^ This perspective aligns with the principle of self-determination,^56^ which emphasizes the importance of autonomy, competence, but also relatedness in motivating behavior change. Therefore, interventions should empower individuals to make autonomous decisions while also providing necessary support and resources. This may be especially relevant for those facing barriers. As will be seen from the findings on the consequences of self-care, some authors recommend integrating self-care with professional care in a balanced manner^57^ to mitigate potential risks such as responsibility overload and professional care neglect.^34^

 Lastly, the analyzed definitions highlight diverse factors that influence self-care, including situational and cultural factors, knowledge and skills, motivation, values, and social interactions (see Table 3). Findings regarding these and other antecedent factors that may facilitate or hinder self-care have undergone a more thorough analysis and are discussed below.

 Together, these recurrent themes suggest that, for the general population, self-care is largely understood as a proactive, goal-oriented process that extends beyond disease management to encompass health and well-being promotion. This has implications for how interventions are framed, for example, by emphasizing strengths and well-being rather than focusing exclusively on risk reduction.

###  Self-Care Antecedents

 To develop effective strategies to promote self-care, it is essential to understand its antecedents. By investigating the factors that precede engagement in self-care, we can identify patterns and determinants that significantly influence the adoption of healthy practices. Knowing the antecedents of self-care allows us to better understand the collective (and individual) needs of a given population. By analyzing the factors that promote or inhibit self-care, we can target specific interventions to meet the unique demands of different groups within the general population.

 Many of the antecedents of self-care identified in the analyzed articles relate to individuals and their internal resources (see Table 4). As previously mentioned, personal factors that either facilitate or hinder self-care include knowledge, skills, motivation, values and beliefs, but also the awareness of the need for self-care, and a sense of responsibility for one’s own well-being. Other relevant factors include psychological (e.g., emotional and cognitive) and demographic (e.g., age) variables. Additionally, factors such as self-efficacy and self-agency are highlighted as playing crucial roles in determining one’s engagement in self-care. Self-efficacy refers to an individual’s belief in their ability to successfully perform a specific task or achieve a particular goal.^58^ As discussed by Richard and Shea,^44^ when it comes to self-care, individuals with high self-efficacy tend to believe they can implement and maintain healthy self-care practices, even when faced with obstacles or difficulties. They feel confident in their ability to manage their physical, emotional, and mental health effectively.^44^ Similarly, Bandura’s^59^ theoryunderscores the role of self-agency and the sense of responsibility in personal well-being, which are pivotal in driving self-care activities.

 Findings on the prerequisites of self-care also highlight that interpersonal and external factors can influence an individual’s ability to engage in self-care (see Table 4), as previously discussed in the definitions of self-care. These factors, which extend beyond the individual, can either facilitate or hinder self-care practices. They include social, spiritual, and professional support systems; access to resources, such as healthcare facilities and information; and the broader social, cultural, and political environment surrounding health. Hence, not only do personal factors serve as antecedents to self-care, but interpersonal, external, cultural, and environmental factors also significantly impact individuals’ ability to engage in self-care. This suggests that influences beyond the individual can either support or hinder self-care practices.^55^

 The interplay of internal factors and external influences on self-care, as evidenced in the analyzed studies, aligns with Bandura’s Social Cognitive Theory.^59^ This theory proposes that human behavior results from a dynamic interaction between personal factors (such as cognitions, emotions, and personality traits, e.g., self-efficacy, motivation), behavioral factors (i.e., individual actions, e.g., exercise, healthy eating), and environmental factors (i.e., external influences from the environment, e.g., social support, access to healthcare). Other approaches, such as the socio-ecological model,^60^ also highlight the impact of multiple levels of influence (i.e., individual, interpersonal, organizational, community, and societal) on self-care behaviors. Understanding the interplay between personal, interpersonal, and external factors is essential for developing comprehensive approaches to promote self-care across different groups within the general population.

 Additionally, understanding the antecedents of self-care allows us to identify potential barriers and obstacles that prevent people from engaging in self-care practices. These may include, for example, lack of access to financial resources or insurance, financial instability, caregiver burden,^45^ limiting beliefs or knowledge about the importance of self-care,^39^ physical limitations, need for social and emotional support,^44^ or policy-related issues within the healthcare system.^20^ By proactively addressing these barriers, we can create more supportive environments for developing healthy and resilient self-care habits. Taken together, the patterns described in this subsection, particularly the emphasis on personal resources (such as self-efficacy, motivation, and a sense of responsibility) alongside repeated references to structural barriers, highlight the need for integrated strategies to promote effective self-care. Such strategies should simultaneously strengthen individuals’ capabilities and modify constraining contexts.

###  Self-Care Consequences/Results

 The results of the review of studies on the consequences of self-care indicate a wide range of benefits (see Table 5). These findings showed that self-care can have significant impacts on different areas of life, both for individuals in the general population (e.g., professionals) and for patients. The learning and development of skills, previously discussed as prerequisites for self-care, are also highlighted as one of the main positive outcomes of self-care. As evidenced in the studies analyzed, self-care increases autonomy and promotes independence, empowering individuals to manage their health conditions. This aligns with Bandura’s^58^ theory of self-efficacy, which states that believing in one’s ability to perform the necessary actions to manage specific situations is crucial for success in various areas of life, including health. Furthermore, self-care can enhance self-esteem and self-confidence, thereby reinforcing this self-efficacy and enabling individuals to adopt more proactive and effective health behaviors.^5^ According to these results, healthcare professionals also benefit from patients’ learning and developing the skills needed for self-care, as this facilitates personalized interventions and improves symptom and disease management. This is consistent with a patient-centered approach, which emphasizes the importance of considering individual needs and preferences in care planning and execution.^61^

 Our findings also indicate that self-care practices have a range of beneficial consequences for the physical and mental health and well-being of individuals, as well as for promoting improvements in lifestyle and overall quality of life, among other broader changes. Literature strongly supports the efficacy of self-care in promoting both mental and physical health, contributing to overall well-being. Adopting healthy habits and making lifestyle changes, central components of self-care, is consistently associated with better health and well-being outcomes.^62^ Deci and Ryan’s ^57^ Self-Determination Theorysupports this perspective, proposing that satisfying basic psychological needs for autonomy, competence, and relatedness fosters individuals’ well-being and mental health.

 Although this is not the focus of our study, the occasional articles that include both patients and the general population seem to indicate beneficial consequences from self-care in the context of disease. This highlights the fundamental role of self-care in disease prevention, detection, and treatment. The ability of individuals to manage their health conditions can reduce morbidity, facilitate recovery, and decrease mortality. Studies such as those by Lorig et al^63^ show that self-care programs for chronic diseases, such as diabetes and arthritis, can significantly improve health outcomes by empowering patients to play an active role in their own care.

 Our study (see Table 5) also highlights the importance of self-care in emotionally demanding professions, with positive outcomes such as reduced occupational stress, burnout, and staff turnover being linked to these practices. This illustrates how empowering these professionals to engage in their self-care practices can decrease burnout and improve job satisfaction, as proposed by Shanafelt et al.^64^ The literature suggests that self-care practices are essential for maintaining resilience and professional effectiveness in challenging work environments.^65^ Furthermore, implementing self-care strategies can improve the quality of patient care and job satisfaction among healthcare professionals, thereby contributing to the sustainability of the healthcare system.

 Overall, the consistent emphasis found across studies on learning and skills development, improvements in health and well-being, disease prevention and management, and reductions in occupational health problems suggest that these outcomes can serve as core markers when evaluating future self-care interventions in the general population. As mentioned earlier in this discussion, although the benefits of self-care are widely recognized, Høy et al.’s^34^ caveat regarding potential adverse effects deserves thoughtful consideration. The risks of an overload of responsibility and possible neglect in professional care should be acknowledged. Therefore, self-care must be integrated with adequate professional care in a balanced manner to ensure that individuals are not left alone to manage complex health conditions.^57^

## Conclusion

 Our literature review on self-care theory, considering its historical origins and evolution, reaffirmed the diversity of the concept of self-care.^3^ Our analysis revealed that the concept of self-care is continuously evolving across various fields of study and for different groups. While the focus on conceptualizing self-care for human services professionalspersists,^7^ other groups within the general population (e.g., categorized by age) are addressed less frequently from a self-care perspective. Future studies should therefore aim to further address this gap. Nevertheless, our review begins to address this by clarifying how self-care in the general population can be understood in terms of its core definitions and attributes, personal and contextual antecedents, and individual and system-level consequences. By organizing these elements into an explicit conceptual model (*cf. *Figure 2), this review provides a shared language and a conceptual framework that can help to inform the selection of constructs and measures, suggest directions for the design of multi-level interventions and policies, and offer a useful reference point for future research on self-care among different groups within the general population, as discussed in more detail below.

 Self-care for the general population, as elucidated by various definitions analyzed, is fundamentally intentional and purpose-driven. It encompasses a wide spectrum of purposes, from managing health conditions to enhancing overall well-being. This multifaceted concept integrates actions, abilities, and socio-cognitive processes, and emphasizes the importance of understanding the multiple antecedents of self-care to develop effective policies and interventions that enhance well-being and quality of life across diverse groups. Recognizing the personal, interpersonal, and external factors that influence self-care can empower individuals to manage their physical, emotional, and psychological health more effectively. Addressing barriers such as financial constraints, limited healthcare access, and cultural influences is also crucial for fostering resilient self-care behaviors. Self-care also requires individual awareness of health status, effective coping strategies, and an understanding of the balance between benefits and barriers. Additionally, social and cultural contexts significantly influence self-care practices, advising tailored interventions that accommodate diverse backgrounds and circumstances. Thus, we argue that the conceptualization of self-care for the general population benefits from a systemic, ecological view.^55,60^ This perspective goes beyond individual-level models by directing research to examine how interactions between personal resources and multilevel contextual factors (e.g., family, schools, workplaces, communities, and health and social policies) enable or constrain self-care, and by informing interventions that combine individual skill-building with changes in social support, service provision, and structural conditions. This approach recognizes the interplay between different factors from multiple systems, enabling the development of comprehensive strategies that address individual needs within broader social and cultural environments.

 The integration of autonomy with external support is crucial for healthy and resilient self-care. This advocates for integrated care approaches that foster sustainable self-care behaviors among individuals in the general population, regardless of their health status. From a systemic, ecological perspective, such approaches require coordinated action across different settings (e.g., linking family, school, community, and health services) to ensure that individuals are supported through consistent messages, resources, and opportunities to practice self-care in their daily lives. From our perspective, the adoption of these self-care behaviors within the broader community can benefit from a promotional, psychoeducational approach targeted at children and young people.^66^ This approach can be achieved through interactions between parents and children, teachers and students, and among children, adolescents, and adults with other community members, including (but not limited to) healthcare professionals. At the same time, it is crucial to provide these agents with training and support to enhance their own self-care while promoting and supporting the self-care of others,^13^ within the framework of broader initiatives. These interventions should be developed through participatory research, also involving children and young people,^67^ particularly in the assessment of self-care needs. These needs may be of varied natures, including regulatory self-care (e.g., eating, sleeping, and personal hygiene), preventive self-care (e.g., exercise, diet, and self-examination), reactive self-care (e.g., self-initiated responses to undiagnosed symptoms), and restorative self-care (e.g., adherence to prescribed medication).^68^

 Moreover, promoting self-care outcomes such as enhanced autonomy, independence, and self-efficacy can lead to benefits such as improved health and well-being for the general population and reduced occupational stress, particularly in high-demand professions. Integrating self-care with comprehensive support systems promotes holistic health management and supports sustainable healthcare practices, benefiting both healthcare providers and individuals, whether they have a health condition or not. In this regard, we appeal to the concepts of ‘collective care’^69^ and ‘collective self-care’,^70^ which emphasize that caring for oneself inherently involves caring for others. This perspective on the dialectical relationship between self-care and caring for others has applications that extend beyond the realm of illness, patients, and healthcare professionals. It may also be relevant for conceptualizing self-care for the general population, including children, youth, adults, and seniors, who are engaged in multiple roles (e.g., students, parents, teachers, citizens) across various life contexts, and who have diverse self-care needs. From this viewpoint, we advocate for a multi-tiered strategy aimed at promoting self-care within the general population, developed through schools, workplaces, and other community contexts. For example, in schools, universal support could entail all students participating in classroom discussions and activities on healthy routines, boundaries, rest, and nutrition. Students showing early signs of fatigue or distress could access targeted interventions, such as small-group self-care workshops, peer mentoring, or check-ins with a school counsellor. Those facing greater challenges could receive individualized support, including personalized self-care plans or ongoing counselling. Similarly, in workplaces, universal self-care initiatives might include encouraging movement breaks, offering healthy snacks, and providing mindfulness sessions. Targeted support for those experiencing stress could involve self-care workshops or peer groups. Individualized counselling or flexible working arrangements could be available for employees facing more acute challenges.

 We argue that this strategy can complement and capitalize on the outcomes of other multi-tiered efforts to promote health and well-being, including social and emotional learning and positive psychology interventions,^66^ or other more traditional public health initiatives.^71^ Therefore, future studies are needed to design, implement, and evaluate the effectiveness and effects of such a strategy. In this context, two key research questions arise for future investigation. Firstly, how do multi-tiered self-care interventions in educational settings (e.g., schools, universities) affect students’ self-care behaviors, well-being, and academic performance across diverse age groups and backgrounds? Secondly, what are the most effective strategies for integrating collective care principles into self-care interventions for adults in workplace or community settings, and how do these influence individual and collective well-being outcomes?

## Strengths and Limitations

 Our study addresses a gap in international literature by contributing to the conceptualization of self-care for the general population, which tends to be overlooked in prior reviews. Nevertheless, our study is not without limitations. To integrate the multiple perspectives found across literature, we adopted a consensual approach, focusing our attention on common baselines. Therefore, we did not delve into contextual specifics, given the extent of the variability and heterogeneity that such an approach would entail. This was necessary given that our literature review included diverse studies regarding target populations, settings, cultural contexts, methodological approaches, and operational definitions. This between-study heterogeneity makes synthesizing the findings challenging and can hinder their overall reliability.^72^ However, by prioritizing areas of conceptual convergence, certain context-specific nuances or less frequently reported elements may have received limited visibility. There is also a risk that isolated or disproportionately emphasized themes, such as ethical considerations, may be given greater prominence in the synthesis than they warrant based on their prevalence in the wider evidence base. Consequently, while our integrative approach provides a high-level overview of the field, between-study heterogeneity reduces the extent to which robust, broad conclusions can be drawn and may increase bias.

 Also, 29% of the articles included in this review were identified through sources other than the main database searches, which is a limitation of this review. While these studies met the eligibility criteria, they did not employ the keywords “conceptualization” or “review”. Instead, they used related terms, such as “definition” or “perspectives”. Expanding the search strategy to include these additional terms would have yielded an unmanageable number of results. Most of these would ultimately be excluded, as the term ‘definition’ was used merely to operationalize self-care in the context of the study, rather than meeting the full eligibility criteria. For the same reason, we also restricted the search terms to the title and/or abstract. Searching for “self-care” and “theory” (and derivatives) in the full text would produce a large volume of low-relevance records and dilute the analytical focus. Thus, our search strategy was optimized to capture studies that explicitly addressed the conceptual underpinnings of self-care, balancing conceptual precision with operational feasibility and maintaining a streamlined and methodologically rigorous screening process. Therefore, although selecting more restrictive search terms and specific search fields may have limited the pool of articles, this was a deliberate decision to maintain the rigor and manageability of the review process.

 Another limitation of our literature review is that conceptual and theoretical studies (37.5% of our record pool) cannot undergo a standard empirical quality assessment. Although we applied a framework to assess conceptual rigor, our evaluation is inherently interpretative and lacks the standardized metrics typically used in empirical quality assessment. This may affect the weighting of evidence and the conclusions that are drawn from these works. Nevertheless, given that our study aimed to explore how self-care can be conceptualized for the general population, we took the decision to prioritize providing an exhaustive range of definitions and did not exclude studies based on quality.

 Moreover, a key finding of our study is that self-care is an ethical imperative and a recognized ethical responsibility of professionals, offering a means of improving their practice.^73,74^ However, despite being identified as an important element in the discussion of self-care, the ethical imperative and its implementation in different societies and professional fields have not been addressed in our study. This is an important issue that should be explored further in future studies. Viewing self-care as an ethical imperative indeed has important implications for future research, particularly in connection with the concepts of ‘collective care’^69^ and ‘collective self-care’.^70^ Self-care is not only a personal and professional responsibility, but also provides a crucial foundation for sustained, competent, and compassionate care for others. From the perspective of collective care, the ethical imperative of self-care involves recognizing that individual well-being directly impacts communities, organizations, and broader social dynamics. Accordingly, future work should address how ethical frameworks around self-care and collective care can be operationalized across professions, cultures, and social groups, moving beyond theory towards practical strategies that foster both individual resilience and collective responsibility for health.

 In sum, given the heterogeneity of study designs and the nature of available research on the conceptualization of self-care, we decided to conduct a literature review. Our primary aim was to take an initial approach to mapping the literature and contributing to an integrated, systematic understanding of how self-care is operationalized for the general population. Nevertheless, given the limitations of our study, it would be valuable for future studies with a narrower scope (e.g., antecedents, ethical issues) to conduct systematic reviews incorporating a quality assessment of the included studies. This would help to strengthen the evidence base in this area.

## Competing Interests

 The authors declare no potential conﬂicts of interest concerning the research, authorship, and/or publication of this article.

## Data Availability Statement

 Not applicable.

## Ethical Approval

 Not applicable.

## Supplementary File

Table S1 - References list containing the 24 studies regarding the final data set of the present study. Table S2 - Systematization of data on quality assessment criteria from eligible studies.
