# Supplementary material for: Toward a Unified Understanding: A Literature Review of Self-Care Conceptualization for the General Population
Source: Health Promot Perspect. 2026 Jun 6;16(1):48–62. doi: 10.34172/hpp.45126 (PMC13402232; doi:10.34172/hpp.45126)
Supplement: Supplementary file 1 — Table S1 - References list containing the 24 studies regarding the final data set of the present study. Table S2 - Systematization of data on quality assessment criteria from eligible studies. [file hpp-16-48-s001.pdf]

Electronic Supplementary Material

Toward a unified understanding: A literature review of self-care conceptualization for the general population

Alexandra Marques-Pinto [1]*, Sofia Oliveira [2], Karina Moutinho [3], Mirela Ricarte [3], Carla Zambaldi [3], Sirley Almeida [4], Pompéia Villachan-Lyra [4], Ana Maria Fonte Alves [4], Taciana Breckenfeld [3], Maria Carolina Marques dos Santos [4], Joana Sampaio de Carvalho [1], & Ari Gómez-Borges [5]

[1] CICPSI, Faculdade de Psicologia, Universidade de Lisboa, Lisbon, Portugal. Emails: ampinto@psicologia.ulisboa.pt (AM-P); joaca73@gmail.com (JSC).
[2] Business Research Unit (BRU), ISCTE - Instituto Universitário de Lisboa, Lisbon, Portugal. Email: sofia.oliveira@iscte-iul.pt (SO)
[3] Universidade Federal de Pernambuco, Recife, PE, Brazil. Email: ana.mlima@ufpe.br (KM), mirela.ricarte@ufpe.br (MR), carla.zambaldi@ufpe.br (CZ), taciana.breckenfeld@ufpe.br (TB)
[4] Universidade Federal Rural de Pernambuco, Recife, PE, Brazil. Email: sa.sirley@gmail.com (SA), pompeia.lyra@ufrpe.br (PV-L), amdafontealves@gmail.com (AMFA), Carolina.msantos04@gmail.com (MCMS)

Table S1

References list containing the 24 studies regarding the final data set of the present study

1.	Bressi, S. K., & Vaden, E. R. (2017). Reconsidering self care. *Clinical Social Work Journal*, *45*(1), 33–38. https://doi.org/10.1007/s10615-016-0575-4 (2)
2.	Chipu, M., & Downing, C. (2020). Professional nurses' facilitation of self-care in intensive care units: A concept analysis. *International Journal of Nursing Sciences*, *7*(4), 446–452. https://doi.org/10.1016/j.ijnss.2020.08.002 (1)
3.	El-Osta, A., Webber, D., Gnani, S., Banarsee, R., Mummery, D., Majeed, A., & Smith, P. (2019). The Self-Care Matrix: A unifying framework for self-care. *Self-Care Adv Study Underst Self-Care, 10*(3), 38-56. (1)
4.	Gantz, S.B. (1990) Self-care: Perspectives from six disciplines. *Holistic Nursing Practice, 4*, 1-12. https://doi.org/10.1097/00004650-199002000-00004 (2)
5.	Gast, H. L., Denyes, M. J., Campbell, J. C., Hartweg, D. L., Schott-Baer, D., & Isenberg, M. (1989). Self-care agency: Conceptualizations and operationalizations. *Advances in Nursing Science*, *12*(1), 26–38. https://doi.org/10.1097/00012272-198910000-00006 (1)
6.	Godfrey, C. M., Harrison, M. B., Lysaght, R., Lamb, M., Graham, I. D., & Oakley, P. (2010). The experience of self-care: A systematic review: *JBI Database of Systematic*

Reviews and Implementation Reports, *8*(34), 1351–1460. https://doi.org/10.11124/01938924-201008340-00001 (2)

7. Godfrey, C. M., Harrison, M. B., Lysaght, R., Lamb, M., Graham, I. D., & Oakley, P. (2011). Care of self–care by other–care of other: The meaning of self-care from research, practice, policy and industry perspectives. *International Journal of Evidence-Based Healthcare, 9*(1), 3-24. https://doi.org/10.1111/j.1744-1609.2010.00196.x (2)

8. Holguín-Lezcano, A., Arroyave-González, L., Ramírez-Torres, V., Echeverry-Largo, W. A., & Rodríguez-Bustamante, A. (2020). O autocuidado como componente da saúde mental do psicólogo na perspectiva biopsicossocial [Self-care as a component of the psychologist's mental health in the biopsychosocial perspective]. *Poiesis, 39*, 149-167. https://doi.org/10.21501/16920945.3760 (2)

9. Horowitz, L. G. (1985). The Self-Care Motivation Model: Theory and practice in healthy human development. *Journal of School Health*, *55*(2), 57–61. https://doi.org/10.1111/j.1746-1561.1985.tb04079.x (2)

10. Høy, B., Wagner, L., & Hall, E. O. C. (2007). Self-care as a health resource of elders: An integrative review of the concept. *Scandinavian Journal of Caring Sciences*, *21*(4), 456–466. https://doi.org/10.1111/j.1471-6712.2006.00491.x (1)

11. Jones, M. C., MacGillivray, S., Kroll, T., Zohoor, A. R., & Connaghan, J. (2011). A thematic analysis of the conceptualization of self-care, self-management and self-management support in the long-term conditions management literature: A conceptual analysis of self-care. *Journal of Nursing and Healthcare of Chronic Illness*, *3*(3), 174–185. https://doi.org/10.1111/j.1752-9824.2011.01096.x (1)

12. Lee, J. J., & Miller, S. E. (2013). A self-care framework for social workers: Building a strong foundation for practice. *Families in Society: The Journal of Contemporary Social Services*, *94*(2), 96–103. https://doi.org/10.1606/1044-3894.4289 (1)

13. Levin, L.S., & Idler, E.L. (1983). Self-care in health. *Annual Review of Public Health*, *4*(1), 181–201. https://doi.org/10.1146/annurev.pu.04.050183.001145(2)

14. Lommi, M., Matarese, M., Alvaro, R., Piredda, M., & De Marinis, M. G. (2015). The evolution of the concept of self-care in the healthcare system: A narrative literature review. *Professioni Infermieristiche*, *68*(2), 155–166. https://doi.org/10.7429/pi.2015.682155 (1)

15. Mailhot, T., Cossette, S., & Alderson, M. (2013). Une analyse évolutionniste du concept d'autosoins. *Recherche en Soins Infirmiers*, *112*(1), 94–106. https://doi.org/10.3917/rsi.112.0094 (1)

16. Martínez, N., Connelly, C. D., Pérez, A., & Calero, P. (2021). Self-care: A concept analysis. *International Journal of Nursing Sciences, 8*(4), 418-425. (1)

17. Marzband, R., & Zakavi, A. A. (2017). A concept analysis of self-care based on Islamic Sources. *International Journal of Nursing Knowledge*, *28*(3), 153–158. https://doi.org/10.1111/2047-3095.12126 (1)

18. Matarese, M., Lommi, M., De Marinis, M. G., & Riegel, B. (2018). A systematic review and integration of concept analyses of self-care and related concepts. *Journal of Nursing Scholarship: An Official Publication of Sigma Theta Tau International Honor Society of Nursing*, *50*(3), 296–305. https://doi.org/10.1111/jnu.12385 (1)

19. McCormack, D. (2003). An examination of the self-care concept uncovers a new direction for healthcare reform. *Nursing Leadership (Toronto, Ont.)*, *16*(4), 48–62. https://doi.org/10.12927/cjnl.2003.16342 (1)

20. Miller, A. E., Green, T. D., & Lambros, K. M. (2019). Foster parent self-care: A conceptual model. *Children and Youth Services Review*, *99*, 107-114. https://doi.org/10.1016/j.childyouth.2019.01.014 (1)

21. Richard, A. A., & Shea, K. (2011). Delineation of self-care and associated concepts: Self-care concept delineation. *Journal of Nursing Scholarship*, *43*(3), 255-264. https://doi.org/10.1111/j.1547-5069.2011.01404.x (1)

22. Tulu, S. N., Cook, P., Oman, K. S., Meek, P., & Kebede Gudina, E. (2021). Chronic disease self-care: A concept analysis. *Nursing Forum*, *56*(3), 734–741. https://doi.org/10.1111/nuf.12577 (1)

23. Wilkinson, A., & Whitehead, L. (2009). Evolution of the concept of self-care and implications for nurses: A literature review. *International Journal of Nursing Studies*, *46*(8), 1143–1147. https://doi.org/10.1016/j.ijnurstu.2008.12.011 (1)

24. Woods, N. (1989). Conceptualizations of self-care: Toward health-oriented models. *Advances in Nursing Science*, *12*(1), 1–13. https://doi.org/10.1097/00012272-198910000-00004 (1)

Note: (1) Studies identified from databases, (2) Studies identified via complementary methods.

Table S2

Systematization of data on quality assessment criteria from the eligible studies.

Reference		Meta-domains				Research design domains			Research conduct domains		Evaluation
**Code**	**Authors (year)**	**Disclosure of research aims**	**Disclosure of funding**	**Disclosure of conflicts of interest**	**Disclosure of authors involvement**	**Research strategy**	**Disclosure of eligibility criteria**	**Theoretical grounding**	**Disclosure of data analysis procedures**	**Disclosure of all findings**	
2	Chipu & Downing (2020)	YES	YES	YES	YES	Concept analysis	YES	NO	NO	YES	Minor concerns
3	El-Osta et al. (2019)	YES	YES	NO	YES	Pragmatic review of literature	YES	YES	YES	YES	No or minimal concerns
6	Godfrey et al. (2010)	YES	YES	YES	NO	Systematic review	YES	YES	YES	YES	No or minimal concerns
7	Godfrey et al. (2011)	YES	NO	NO	NO	Literature review	YES	NO	YES	YES	Moderate concerns / Unclear
8	Holguín-Lezcano et al. (2020)	YES	YES	YES	NO	Document analysis	NO	YES	YES	YES	Minor concerns
10	Høy et al. (2007)	YES	YES	NO	YES	Integrative review	YES	NO	YES	YES	Minor concerns
11	Jones et al. (2011)	YES	YES	NO	YES	Iterative literature search	YES	NO	YES	YES	Minor concerns
14	Lommi et al. (2015)	YES	NO	NO	NO	Narrative literature review	YES	YES	YES	YES	Minor concerns
15	Mailhot et al. (2013)	YES	NO	NO	NO	Concept analysis	YES	YES	YES	YES	Minor concerns
16	Martínez et al. (2021)	YES	YES	YES	YES	Concept analysis	YES	YES	YES	YES	No or minimal concerns
17	Marzband & Zakavi (2017)	YES	YES	YES	YES	Concept analysis	YES	NO	YES	YES	No or minimal concerns
18	Matarese et al. (2018)	YES	NO	NO	YES	Systematic review	YES	YES	YES	YES	No or minimal concerns

21	Richard & Shea (2011)	YES	NO	NO	NO	Literature review	NO	YES	YES	YES	Moderate concerns / Unclear
22	Tulu et al. (2022)	YES	NO	NO	NO	Concept analysis	YES	NO	NO	YES	Moderate concerns / Unclear
23	Wilkinson & Whitehead (2009)	YES	NO	YES	NO	Literature review	YES	YES	NO	YES	Minor concerns

Note. Criteria and coding adapted from CAMELOT-informed assessment (Munthe-Kaas et al., 2024).
